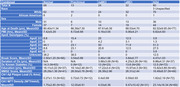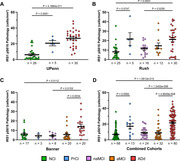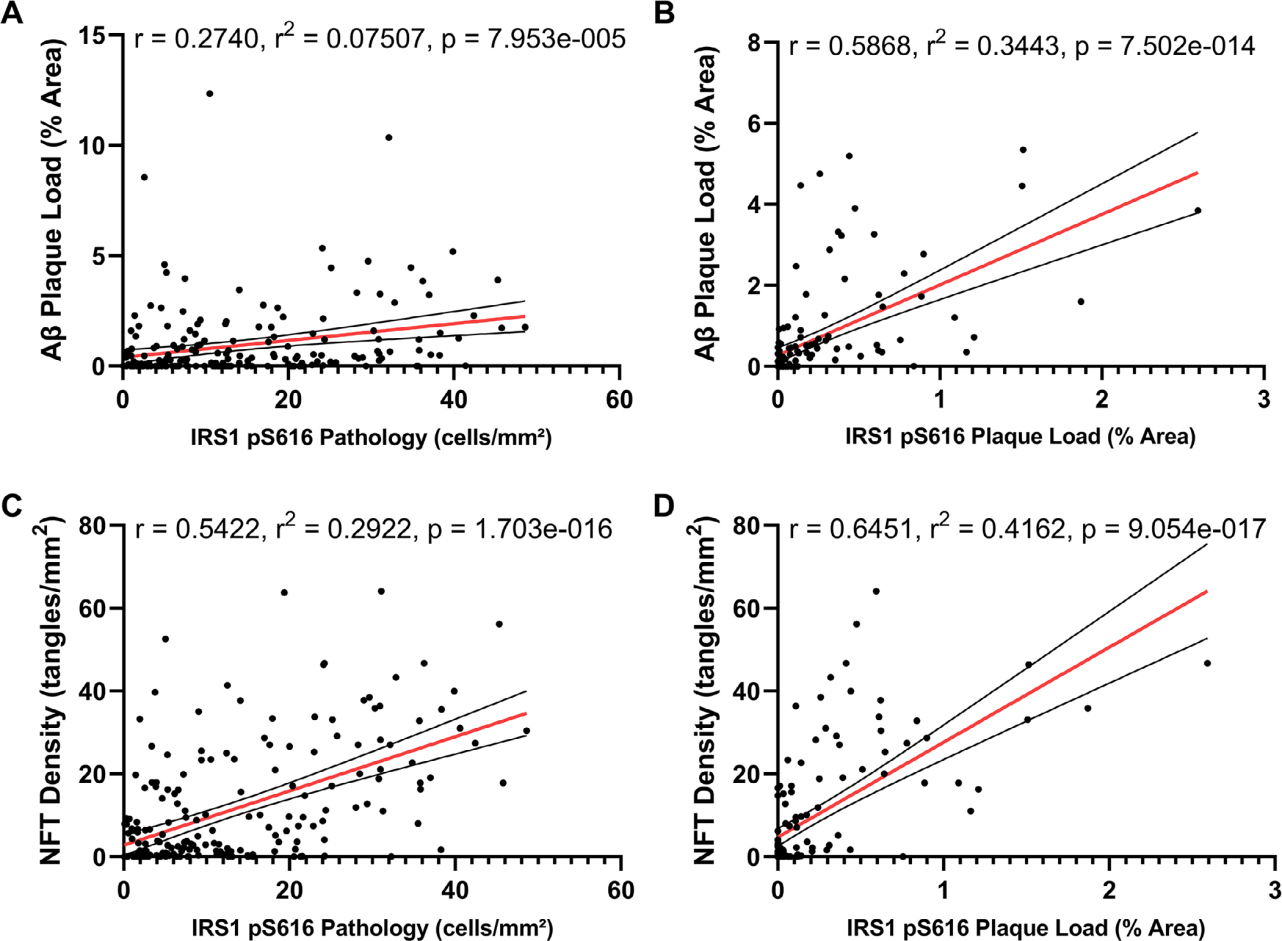# Elevated IRS‐1 pS616 Pathology in Human Hippocampal Area CA1 as a Biomarker of Preclinical and Alzheimer's Disease Dementia

**DOI:** 10.1002/alz.093219

**Published:** 2025-01-09

**Authors:** Xuelin Gu, Tim Distel, Konrad Talbot

**Affiliations:** ^1^ Loma Linda University, Loma Linda, CA USA; ^2^ Loma Linda University Health, Loma Linda, CA USA

## Abstract

**Background:**

Alzheimer's disease (AD) is a progressive neurodegenerative disease whose risk can be assessed in the AT(N) framework based on brain levels of Aβ and pathological tau with or without neuronal injury. This helps determine if a cognitively normal or mildly cognitively impaired (MCI) person has clear signs of AD pathogenesis. The AT(N) framework might be enhanced by also considering brain insulin resistance (BIR), which is a common feature in AD dementia (ADd). However, a clear biomarker for brain insulin resistance has yet to be identified. Some studies have identified elevated insulin receptor substrate‐1 phosphorylated at S616 (IRS‐1^pS616^) in ADd, with conflicting results in other studies. Here, we observed IRS‐1^pS616^ is mostly limited to cell nuclei in hippocampal field CA1 of normal cases but becomes prominent in neuronal cytoplasm in ADd cases, quantifying this pathological feature using artificial intelligence‐based analysis.

**Methods:**

We performed quantitative immunohistochemical studies on fixed hippocampal sections from 217 age‐ and sex‐matched cases: 68 non‐cognitively impaired (NCI), 13 preclinical (PrCl; i.e., NCI but A+T+), 24 non‐amnestic MCI (naMCI), 32 amnestic MCI (aMCI), and 80 ADd from 3 brain banks (University of Pennsylvania [UPenn], Rush University, and the Banner Research Institute) for β‐amyloid (NAB228), phospho‐tau (AT8), and IRS‐1^pS616^ (44‐550G). Immunoreactive cells and plaques were quantified using supervised deep‐learning U‐Net neural networks with additional morphology‐based criterions.

**Results:**

Demographical characteristics of study participants are presented in Table 1. The density of CA1 neurons with IRS‐1^pS616^ pathology was significantly elevated in ADd cases across all cohorts, in PrCl cases from some cohorts, but not in MCI cases (Figure 1). Significant associations were found between IRS‐1^pS616^, β‐amyloid, and phospho‐tau pathologies in regression analyses, which were weak between IRS‐1^pS616^ cells and β‐amyloid plaques (Figure 2). No significant differences in IRS‐1^pS616^ pathology were found between APOE genotypes.

**Conclusion:**

These results suggest that elevated IRS‐1^pS616^ pathology in hippocampal field CA1 is a potential biomarker for BIR in preclinical and ADd, which may be treatable with antidiabetics in the class of incretin receptor agonists shown to reduce BIR in ex vivo preparations of the hippocampus from MCI and ADd cases (see Abstract 89821).